# Crosstalk between keratinocytes and neutrophils shapes skin immunity against *S. aureus* infection

**DOI:** 10.3389/fimmu.2024.1275153

**Published:** 2024-02-16

**Authors:** Jule Focken, Birgit Schittek

**Affiliations:** ^1^ Department of Dermatology, University Hospital Tübingen, Tübingen, Germany; ^2^ Cluster of Excellence 2124 Controlling Microbes to Fight Infections, University of Tübingen, Tübingen, Germany

**Keywords:** neutrophils, keratinocytes, *Staphylococcus aureus*, skin inflammation, skin immune system

## Abstract

**Introduction:**

*Staphylococcus aureus* (*S. aureus*) infection of the skin leads to a rapid initial innate immune response with keratinocytes in the epidermis as the initial sensors. Polymorphonuclear neutrophils (PMNs) are the first innate immune cells to infiltrate infection sites where they provide an effective first-line of defense. Previous work of our group showed that in inflamed skin a crosstalk between PMNs and keratinocytes results in enhanced *S. aureus* skin colonization.

**Methods:**

In this work, we used an *in vitro* co-culture model to studied the crosstalk between primary human keratinocytes (PHKs) and PMNs in a sterile environment and upon S. aureus infection. We investigated the influence of PHKs on PMN activation by analyzing PMN lifespan, expression of degranulation markers and induction of proinflammatory cytokines. Furthermore, we analyzed the influence of PMNs on the inflammatory response of PHKs. Finally, we investigated the influence of the skin microbiome on PMN-mediated skin inflammation.

**Results:**

We show that co-culture of PMNs with PHKs induces activation and degranulation of PMNs and significantly enhances their lifespan compared to PMN cultivation alone by an IL-8 mediated mechanism and, furthermore, primes PMNs for enhanced activity after *S. aureus* infection. The prolonged incubation with PMNs also induces inflammatory responses in PHKs which are further exacerbated in the presence of *S. aureus* and induces further PMN recruitment thus fueling skin inflammation. Interestingly, infection of PHKs with the skin commensal *S. epidermidis* reduces the inflammatory effects of PMNs in the skin and exhibits an anti-inflammatory effect.

**Discussion:**

Our data indicate that skin infiltrating PMNs and PHKs influence each other in such a way to enhance skin inflammation and that commensal bacteria are able to reduce the inflammatory effect.

## Graphical Abstract

Proposed model of the interplay between PMNs and PHKs during skin inflammation. In a sterile environment, PMNs undergo activation, with their lifespan extended through an IL-8 dependent way. Concurrently, proinflammatory responses are initiated in PHKs. Skin infection with *S. aureus* intensifies inflammation, leading to enhanced recruitment of PMNs. Conversely, *S. epidermidis* exerts an anti-inflammatory influence by mitigating PMN-mediated skin inflammation and facilitating apoptosis in PMNs. This illustration was created with BioRender.

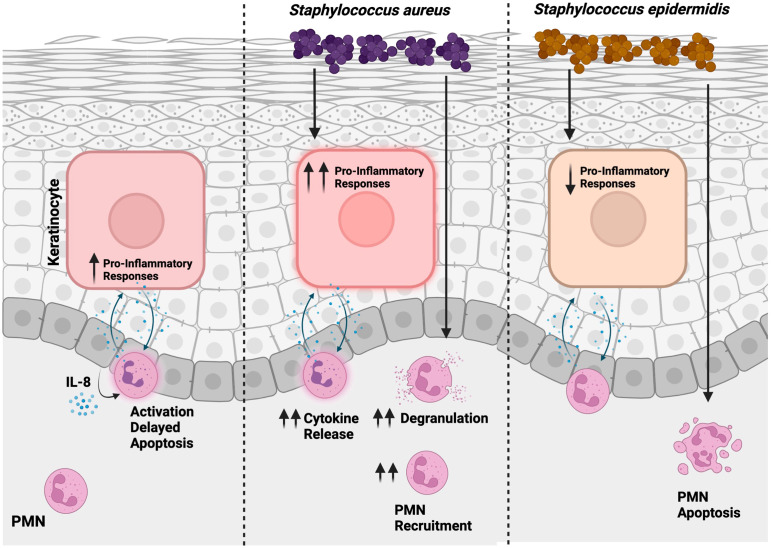

## Introduction

The skin immune barrier depends on the interplay of different cell types to ensure homeostasis under physiological conditions and protection against invading pathogens (1). Keratinocytes are the main constituents of the epidermis and thus the first cells to sense an invading pathogen such as *Staphylococcus aureus* (*S. aureus*) and therefore play a crucial role in initiating and maintaining skin inflammation (2). They contain pattern-recognition receptors that help to sense pathogen-associated-molecular-patterns (PAMPs) on the microbes which initiates secretion of cytokines, chemokines, and antimicrobial peptides (AMPs) and the recruitment of immune cells to the site of infection (2).

Polymorphonuclear neutrophils (PMNs) are the most abundant leucocytes in the human blood (3). Upon skin infection, they are the first cells to infiltrate the infected site where they provide an effective first-line of defense (4, 5). To ensure a fast response at infection sites, PMNs contain preformed molecules stored in cytoplasmic granules that can be rapidly mobilized via degranulation (6, 7). However, excessive degranulation can cause enormous collateral damage to surrounding tissue and lead to systemic inflammation. Therefore, PMN activation and degranulation needs to be tightly controlled and requires receptor-coupled mechanisms (6). After completing their tasks, PMNs undergo apoptosis and are cleared by macrophages. This prevents excessive inflammation and helps restoring homeostasis (8–10).

*S. aureus* is a gram-positive facultative pathogen responsible for the majority of skin infections in humans. It asymptomatically colonizes about 30% of the anterior nares of the human population (11, 12). *S. aureus* can be frequently found on the skin of atopic dermatitis patients where it actively contributes to skin inflammation (13, 14). It has been shown that upon *S. aureus* infection of the skin, the recruitment of PMNs is critical in clearing the infection (15, 16). However, we and others show that the presence of PMNs in the skin of mice or in a human *in vitro* coculture system of PHKs and PMNs enhances *S. aureus* skin colonization and persistence by the interaction of PHKs with neutrophil extracellular traps (NETs) (17, 18). These data indicate that an interaction of PHKs with PMNs via NETs are important to keep infections local in the initial stages of colonization. Further studies of us revealed that PMNs and NETs present in the inflamed skin induce oxidative stress in PHKs which results in the secretion of HMGB1. NETs and HMGB1 can downregulate the expression of epidermal barrier genes thus promoting skin barrier defects which favors *S. aureus* skin colonization (19).

Here, we studied the crosstalk between keratinocytes and PMNs independent of NETs in a sterile and an infectious environment. We investigated the influence of PHKs on activation of PMNs by analyzing PMN lifespan, expression of degranulation markers and induction of proinflammatory cytokines. Vice versa, we analyzed the influence of PMNs on the inflammatory response of PHKs. Finally, we investigated the influence of the skin microbiome on PMN-mediated skin inflammation. Our data highlight a sophisticated crosstalk of PMNs with PHKs in the skin which shapes immune responses to invading bacteria.

## Materials and methods

### Isolation of primary human PMNs

Peripheral blood was drawn from healthy donors, mixed with dextran solution (2% Dextran, 0.9% NaCl) and incubated for 30 min at RT. The upper phase was layered onto BioColl (1.077 g/ml, Bio&Sell) in a 3:2 ratio and density gradient centrifugation was performed for 30 min at 1600 rpm in a swinging bucket rotor without brake. The cell pellet was resuspended in hypotonic erythrocyte lysis buffer (C-C-Pro). After 10 min incubation time, a second centrifugation step at 1600 rpm for 10 min without brake was performed. After one washing step with PBS, the remaining cell pellet containing the PMNs was resuspended in keratinocyte base medium (CELLnTECH) containing 1.7 mM CaCl_2_. PMN isolation from human blood was approved by the ethics committee of the medical faculty of the University of Tübingen (054/2017BO2).

### Cell culture and *in vitro* co-culture system

Primary human keratinocytes (PHKs) isolated from human foreskin after routine circumcision from the Loretto Clinic in Tübingen as previously described (18, 20, 21). PHK isolation was approved by the ethics committee of the medical faculty of the University of Tübingen (654/2014BO2) and performed according to the principles of the Declaration of Helsinki. PHKs were cultured in collagen-coated tissue flasks (Corning, BioCoat™) in epidermal keratinocyte medium (CELLnTEC) at 37°C, 5% CO_2_. 24h before the experiments, PHKs were differentiated with keratinocyte base medium (CELLnTECH) containing 1.7 mM CaCl_2_. The *in vitro* co-culture model was performed as previously described (18). Briefly, PHKs were seeded into collagen-coated transwell inserts (pore size: 0.4 µm) and differentiated after reaching confluency. PMNs were isolated as described above and seeded into 24 well plates in a concentration of 2x10^6/ml. The inserts containing differentiated PHKs were placed on top of the PMNs, and the cells were co-incubated for the indicated times.

### Flow cytometry

1x10^6 PMNs were incubated with the following surface antibodies for 20 min on ice in the dark. Surface antibodies included PerCP-Cy5.5-anti-CD11b, APC-anti-CD63, APC-anti-CD66b (all Biolegend). To exclude dead cells, a fixable viability dye was included. Flow cytometry was performed using an LSR II (BD Bioscience) and analyzed with FlowJo (TreeStar).

### Annexin-V staining

Annexin-V staining was performed as previously described. Briefly, 1x10^6 PMNs were resuspended in Annexin binding buffer containing Annexin and incubated for 15 min at RT in the dark. After one washing step with annexin binding buffer, PMNs were measured at the LSR II (BD Bioscience) and analyzed with FlowJo (TreeStar).

### Live cell imaging

The Incucyte SX1(Sartorius) was used to investigate PMN viability over time. PMNs were seeded in a 24 well plate in a concentration of 1x10^6/ml and were either co-incubated with PHKs or stimulated with the PMN supernatant of the 18h co-culture. Non-co-cultured PMNs or PMNs incubated with medium served as control. DRAQ5 (1 uM; Thermofisher) was used for staining the intracellular DNA and Sytox Green (0.2 uM; Thermofisher) was used for staining the extracellular DNA, thus indicating cell death. Pictures were taken every hour when PMNs were stimulated with the supernatant or after 6h, 18h and 30h when co-cultured with PHKs. Percentage of Sytox Green positive cells were quantified using Fiji/ImageJ.

### Western blot

Analysis of capase-3 cleavage in PMNs was performed by western blot using whole cell lysates. Briefly, PMNs were lysed in a lysis buffer containing protease and phosphatase inhibitors. Lysates were separated via SDS-polyacrylamide gel electrophoresis and plotted onto PVDF membranes. After 60 min blocking in PBS + 01% Tween + 5% dry milk, membranes were incubated in anti-caspase-3 antibody (1:1000, Cell Signaling) at 4°C overnight. The next day, the membrane was washed three times with PBS + 0.1% Tween before incubation in a secondary antibody, a horseradish peroxidase-conjugated anti-rabbit IgG (1:2000, Cell Signaling). ECL (Thermo Fisher Scientific) was used as chemiluminescence reagents, and an Amersham Imager 600 (General Electric) was used for detection.

### LEGENDplex™ multiplex cytokine analysis

Cytokine analysis was performed with 25 μl of cell culture supernatants of either the PHK well or the PMN well of the co-culture using the LEGENDplex™ human cytokine panel 2 and essential immune response panel (BioLegend) according to the manufacturer’s instructions. Samples were acquired using a LSRII flow cytometry (BD Biosciences) and analyzed using the LEGENDplex™ Software (BioLegend). We assured that the cytokines we detect in the respective wells are derived from PMNs or PHKs, respectively.

### Enzyme-linked immunosorbent assay

Secreted MPO and LCN2 in cell culture supernatants were analyzed using DuoSet ELISA Kits from R&D according to the manufacturer’s instructions. Briefly, ELISA plates (Nunc) were coated with 50 μl capture antibody overnight at 4°C. The next day, the plate was washed three times with PBS + 0.05% Tween before being incubated in PBS + 1% BSA for 1h at RT. After three washing steps with PBS + 0.05% Tween, 50 μl cell culture supernatant or standards were added, and the plate was incubated for 2h at RT. The plate was washed three times and incubated with a biotinylated detection antibody for 2h at RT. After three washing steps, the plate was incubated in HRP-conjugated streptavidin for 20 min at RT in the dark. The plate was subsequently washed and a TMB substrate solution (Cell Signaling) was added to each well. The reaction was stopped with 2N H_2_SO_4_ and absorbance at 450 nm was measured using a Fluoroskan II (Labsystems).

### RNA isolation & cDNA generation

Total RNA isolation of PHKs was performed using the RNA kit (Macherey-Nagel) according to the manufacturer’s protocol. Following RNA isolation, complementary DNA was synthesized using the Reverse-Transcriptase Kit (Thermo Scientific) with 1 μg of RNA, 4 μl of 5x RT buffer, 0.5 μl Maxima reverse transcriptase (200 U/ml), 1 μl of random hexamer primer (100 μM), dNTP (1mM) and RNAse-free water to a total volume of 20 μl. RNA was first pre-incubated with RNAse-free water at 70°C for 10 min before the master mix was added and cDNA was synthesized for 10 min at 25°C and 45 min at 50°C followed by a heat-inactivation step for 5 min at 85°C.

### RT^2^ Profiler™ PCR Array

RT^2^ Profiler™ PCR Array Antibacterial Response (PAHS-148Z) was used for the analysis of genes involved in inflammation and immune responses in PHKs after 18h co-incubation with PMNs. As control, non-co-cultured PHKs were used. The assay and subsequent data analysis was performed according to the manufacturer’s instructions. Samples were acquired in triplicates.

### Bacterial strains

*Staphylococcus aureus* USA300 LAC and *Staphylococcus epidermidis* 1457 was used in this study. Bacteria were aerobically grown in tryptic soy broth (TSB) at 37°C and orbital shaking. All experiments were performed with logarithmically grown bacteria (OD = 0.5).

### Neutrophil recruitment assay

For the PMN recruitment assay, freshly isolated PMNs were labeled with 1 μM Calcein (eBioscience) for 30 min, washed and seeded into a 3 μm transwell insert (Sarstedt). The transwell insert was then placed above a well containing different stimuli. After 1h, the inserts were removed and PMNs migrated into the lower well were lysed using 1% Triton X-100. The fluorescence of the lysates was quantified in triplicates using a Fluoroskan II (Labsystems). As stimuli, the supernatants of the PHK well (upper well) or the PMN well (lower well) of the 18h co-culture with or without *S. aureus* infection was used. As controls, the supernatants of non-co-cultured PHKs with and without *S. aureus* infection and non-co-cultured PMNs was included. As positive control, N-formyl-met-leu-phe was used (fMLF). Prior to the migration assay, the supernatants were centrifuged, and filter sterilized. To calculate the absolute numbers of migrated PMNs, a standard curve was included.

### Statistical analysis

Significant differences between the means of the different treatments were evaluated using GraphPad Prism 9.0 (GraphPad Software, Inc.). Either unpaired, two-tailed Student’s t test, one-way analysis of variance followed by Dunnett’s multiple comparisons test or two-way analysis of variance followed by Šídák’s multiple comparisons test was used for statistical analysis and indicated in the respective figure legends. Differences were considered statistically significant with a p value of <0.05. Data were visualized using GraphPad 9.0 (GraphPad Software Inc.), MS Excel (Microsoft Corporation), FlowJo (TreeStar) or Fiji/ImageJ.

## Results

### Co-incubation with PHKs prolongs the lifespan of PMNs

**Figure 1:**
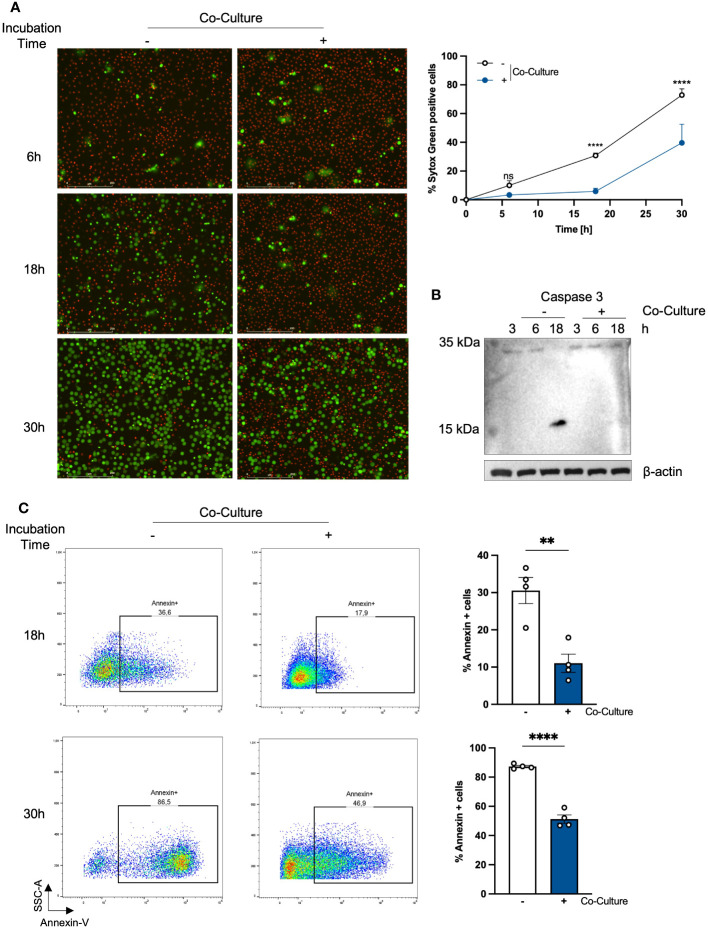
Co-incubation with PHKs prolongs the lifespan of PMNs. **(A)** PMNs (2x10^6/ml) were either incubated alone or in co-culture with differentiated PHKs seeded in a transwell insert (pore size 0.4 μm) and placed above the PMNs. Cell viability was analyzed at different times by quantifying the percentage of Sytox Green positive cells (n = 4). Representative pictures of one experiment after 6h, 18h and 30 are shown. Scale bars = 200 μm. Significant differences between non-co-cultured and co-cultured PMNs at the different time point were analyzed by two-way ANOVA *P < 0.05, **P < 0.01, ***P < 0.001, ****P < 0.0001. Shown are the mean of four different experiments + SEM. **(B)** Induction of apoptosis in non-co-cultured and co-cultured PMNs was analyzed at 3h, 6h and 18h incubation time by investigating caspase-3 cleavage via western blot. Protein expression of β-actin was used as loading control. **(C)** Apoptosis induction in non-co-cultured and co-cultured PMNs was analyzed at 18h and 30h by Annexin-V staining. Annexin positive cells were quantified and represent apoptotic cells. Shown are the mean of four different experiments + SEM. Significant differences were analyzed by unpaired two-tailed t-tests *P < 0.05, **P < 0.01, ***P < 0.001, ****P < 0.0001. On representative experiment of three independent experiments is shown. PHKs, primary human keratinocytes; PMNs, polymorphonuclear neutrophils; SEM, standard error of the mean.

First, we were interested whether the viability of PMNs changes when we co-cultured with PHKs. Therefore, we analyzed the viability of PMNs by SYTOX Green staining, a non-permeable dye indicating dead cells, at different time points after co-culture with PHKs. We used an established *in vitro* co-culture transwell chamber model (18) and compared it to the results of the non-co-cultured PMNs (**Figure 1A**). We found that the percentage of SYTOX Green positive cells steadily increases overtime in the non-co-cultured PMNs. In contrast to this, co-cultured PMNs exhibit a significantly extended lifespan, with only little increase in Sytox Green positive cells in the first 18h. However, the induction of cell death was not completely prevented in co-cultured PMNs as we see a clear increase in SYTOX Green positive cells at 30h co-incubation time. Therefore, we conclude that the co-culture with PHKs significantly delays the induction of cell death in PMNs. We calculated the delay of cell death induction between the co-cultured and non-co-cultured PMNs using interpolation and found a delay of 9.3h until 50% of the cells are dead (**Supplementary Figure 1A**). To unravel the type of cell death, we analyzed the induction of apoptosis in co-cultured and non-co-cultured PMNs by caspase-3 cleavage and Annexin-V staining (**Figures 1B, C**). After 18h, apoptosis induction of non-co-cultured PMNs was indicated by cleaved caspase-3 which was not observed in co-cultured PMNs (**Figure 1B**). Furthermore, we used Annexin-V staining, which detects cells in the early or late apoptosis phase before the cells lose their membrane integrity and get Sytox positive. Interestingly, Annexin-V staining after different incubation times revealed that apoptosis induction was significantly delayed in co-cultured PMNs compared to non-co-cultured PMNs (**Figure 1C**) confirming our Sytox experiments. Together, these data indicate that in the co-culture a beneficial interaction between PHKs and PMNs significantly extend the viability of PMNs by delaying apoptosis induction.

### The prolonged lifespan of PMNs in the co-culture with PHKs is mediated by secreted IL-8

**Figure 2:**
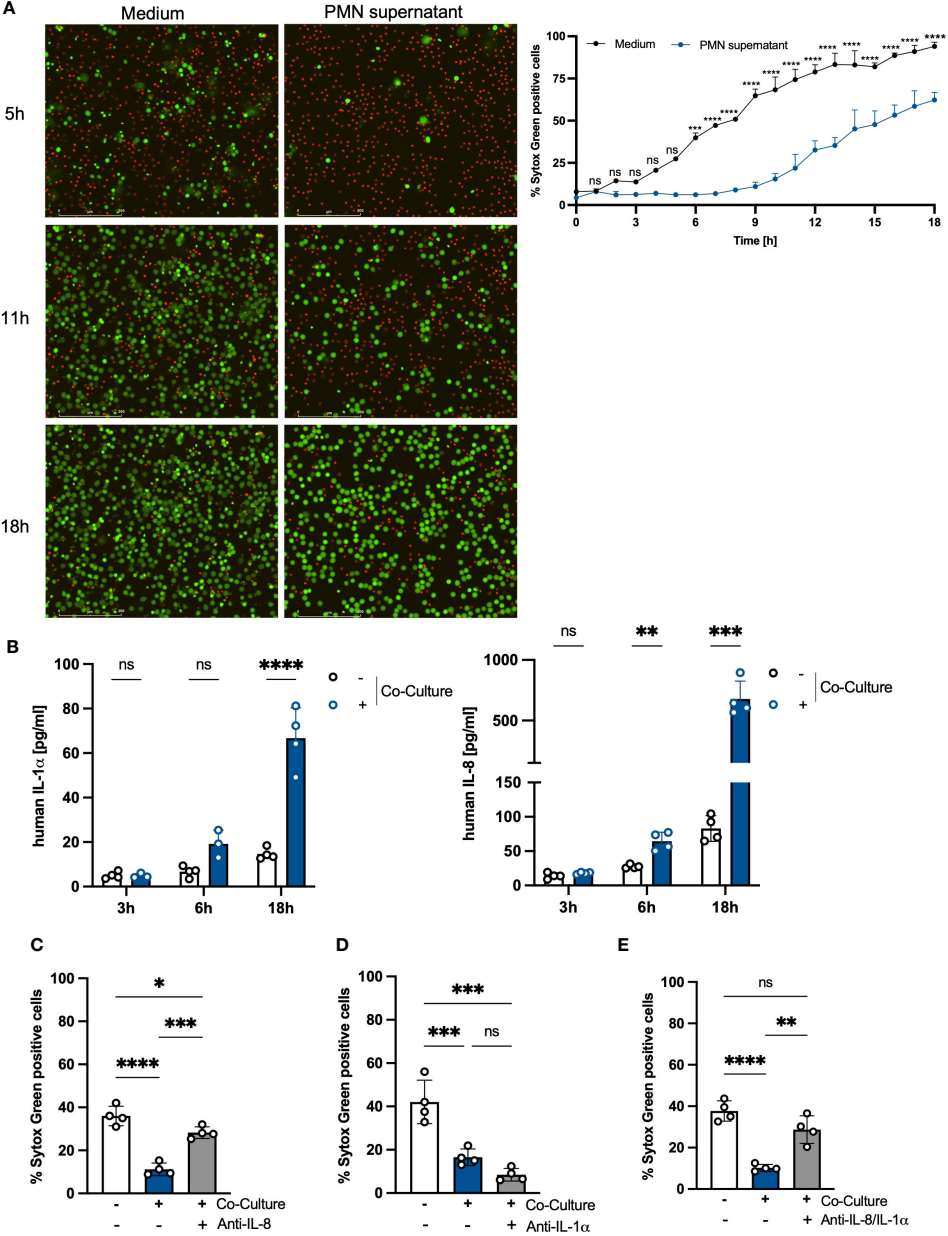
The prolonged lifespan of PMNs in the co-culture with PHKs is partly mediated by secreted IL-8. **(A)** Freshly isolated PMNs (2x10^6/ml) were incubated either in medium or in the PMN supernatant of the 18h co-culture. Cell viability was analyzed over time by Sytox Green staining and live cell imaging. The percentage of Sytox Green positive cells, indicating dead cells, was quantified every hour for 18h using Fiji/ImageJ (n = 3). Representative images of the same cell area after 5h, 11h and 18h incubation are shown. Scale bars = 200 μm. **(B)** Freshly isolated PMNs were co-cultured with differentiated PHKs or alone. After 3h, 6h and 18h, secreted factors in the PMN well were analyzed using Legendplex analysis. Significant differences between the non-co-cultured and co-cultured PMNs were analyzed for each time point by multiple unpaired t-tests *P < 0.05, **P < 0.01, ***P < 0.001, ****P < 0.0001. Shown is one representative experiment of four independent experiments + SD. **(C–E)** PMNs were either incubated alone or in co-culture with differentiated PHKs in the presence or absence of an anti-IL-8 antibody **(C)** or anti-IL-1α antibody **(D)** or both **(E)**. Induction of cell death was analyzed after 18h by quantifying Sytox Green positive cells using Fiji/Image J (n = 4). Shown is one representative experiment of four independent experiments + SD. Significant differences were analyzed between the percentage of SYTOX Green positive cells by one-way ANOVA *P < 0.05, **P < 0.01, ***P < 0.001, ****P < 0.0001. PMNs, polymorphonuclear neutrophils; PHKs, primary human keratinocytes; IL-1α, interleukin 1α; IL-8, interleukin 8; SD, standard deviation. ns, non significant.

We hypothesized that the delayed apoptosis induction in PMNs is mediated by soluble factors released by the PMNs itself during the co-incubation with PHKs. To test this hypothesis, we co-cultured PMNs with PHKs for 18h and subsequently collected the supernatant of the PMN well, filter-sterilized it and used it for stimulation of freshly isolated PMNs. Non-co-cultured PMNs incubated in medium were used as control. We investigated cell death of PMNs over time by Sytox Green staining and live cell imaging (**Figure 2A**). While the percentage of Sytox-Green-positive cells increased significantly after 5 hours in control PMNs cultured in medium, the induction of cell death was delayed in PMNs incubated with PMN supernatant. Here, a significant increase in Sytox-Green-positive cells was observed after 11 hours, which then steadily increased over time. We calculated the delay in cell death induction between PMNs incubated in medium or PMN supernatant by interpolation and found a delay of 8.3 hours until 50% of the cells were dead (**Supplementary Figure 1B**). This delay (8.3 hours) is comparable to the delay found in co-cultured *vs* non-co-cultured PMNs (9.3 hours), observed in **Figure 1A** which indicates that the soluble factors released by PMNs during the co-culture are mainly responsible for the extended lifespan. To identify these factors, we performed LEGENDplex analysis of the PMN well after 3h, 6h and 18h co-culture with PHKs and compared these to secreted factors from non-co-cultured PMNs. We detected significantly increased amounts of secreted Interleukin (IL)-8 and IL-1α in PMNs co-cultured with PHKs compared to non-co-cultured PMNs after 18h (**Figure 2B**). Other examined cytokines were not significantly induced (**Supplementary Figure 2A**). To test whether the IL-1α and IL-8 are responsible for the enhanced viability of PMNs co-cultured with PHKs, we co-cultured PMNs with PHKs for 18h in the presence of absence of either an anti-IL-8, anti-Il-1α or both antibodies together and analyzed cell viability by Sytox Green staining. We compared the results to the non-cocultured PMNs. Interestingly, addition of the anti-IL-8 antibody led to a significant reversal of the life-prolonging effect of the co-culture, whereas IL-α had no significant life-prolonging effect (**Figures 2C, D**; **Supplementary Figures 2B, C**). Interestingly, the combined treatment with an anti-IL8 and anti-IL-1α antibody still reverted the life-prolonging effect of the co-culture indicating that the effect of IL-8 dominates compared to IL-1α in the life prolongation of PMNs in the co-culture (**Figure 2E**; **Supplementary Figure 2D**). Interestingly, recombinant IL-8 delayed apoptosis in freshly isolated PMNs in a concentration-dependent manner, further highlighting the anti-apoptotic properties of IL-8 (**Supplementary Figure 2E**).

### Co-culture with PHKs activates PMNs

**Figure 3:**
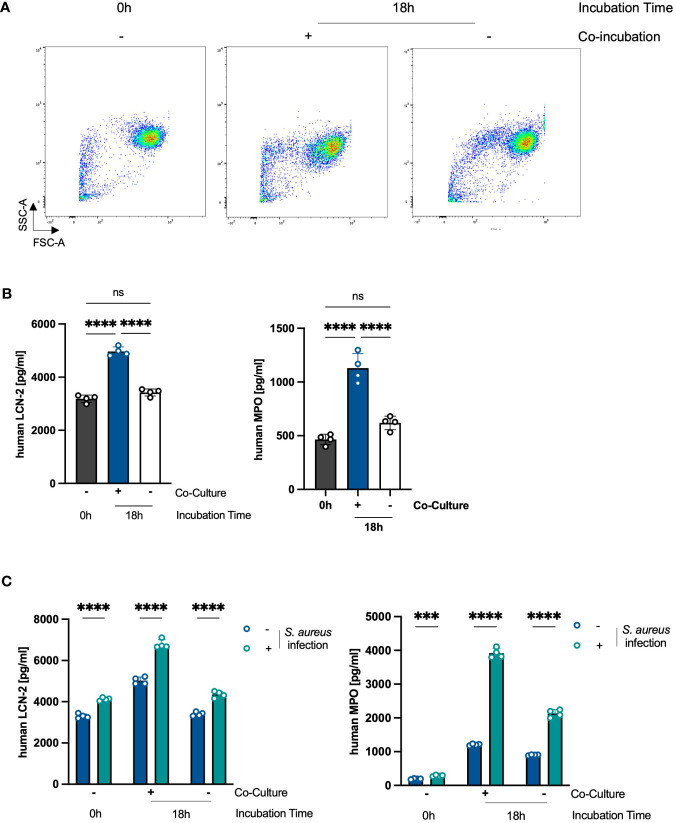
Co-Culture with PHKs activates PMNs. **(A)** Forward Scatter (FSC) and Side Scatter (SSC) of freshly isolated PMNs and PMNs co-cultured with differentiated PHKs or alone for 18h were analyzed by flow cytometry. **(B)** Secreted levels of MPO and LCN-2 by co-cultured and non-co-cultured PMNs after 18h incubation was analyzed by ELISA and compared to freshly isolated PMNs (0h). Shown is one representative experiment of three independent experiments + SD. Significant differences were analyzed by one-way ANOVA *P < 0.05, **P < 0.01, ***P < 0.001, ****P < 0.0001. One representative experiment of three independent experiments is shown. **(C)** Freshly isolated (0h), 18h co-cultured and 18h non-co-cultured PMNs were infected directly with *S. aureus* (MOI = 10) or left uninfected. After 2h, secreted MPO and LCN2 levels were analyzed by ELISA. Significant differences between uninfected and infected PMNs were analyzed by unpaired two-tailed t-tests *P < 0.05, **P < 0.01, ***P < 0.001, ****P < 0.0001. Shown is one representative experiment of three independent experiments + SD. PMNs, polymorphonuclear neutrophils; PHKs, primary human keratinocytes; MOI, multiplicity of infection; MPO, myeloperoxidase; LNC2, lipocalin-2. ns, non significant.

We next analyzed whether along with extending their lifespan, the co-culture with PHKs activates PMNs. Activated PMNs are characterized by degranulation (22). Indeed, we observed a decrease in the side scatter (SSC) of co-cultured PMNs after 18h compared to non-co-cultured PMNs or freshly isolated PMNs (0h), indicative for reduced granularity and thus degranulation (**Figure 3A**). We further analyzed degranulation by analyzing surface expression of CD11b, CD66b and CD63 as well as extracellular levels of MPO and LCN2, all markers for degranulation. We observed that PMNs co-cultured with PHKs for 18h released significantly more MPO and LCN2 compared to non-co-cultured PMNs or freshly isolated PMNs (**Figure 3B**). Furthermore, while we did not detect a significant difference in the surface expression of CD11b or CD66b, surface expression of CD63 was significantly increased in co-cultured PMNs compared to non-co-cultured or freshly isolated PMNs (**Supplementary Figure 3**). Together, these data indicate that PMNs are in an activated state after co-culture with PHKs.

We next hypothesized that already activated state leads to an enhanced activation of PMNs in response to an infectious stimulus. To investigate this, we infected freshly isolated PMNs or PMNs non-co-cultured or co-cultured with PHKs for 18h with *S. aureus* (MOI = 10) and analyzed PMN activation by the analysis of extracellular levels of MPO and LCN2 as markers for degranulation. Interestingly, while *S. aureus* infection resulted in increased levels of secreted MPO and LCN2 in all conditions, the increase in MPO and LCN2 levels was especially prominent in co-cultured PMNs (**Figure 3C**). Together, these data indicate that the co-culture with PHKs primes PMNs for enhanced activation in response to an infectious stimulus.

### Co-incubation with PMNs activates an inflammatory response in PHKs

**Figure 4:**
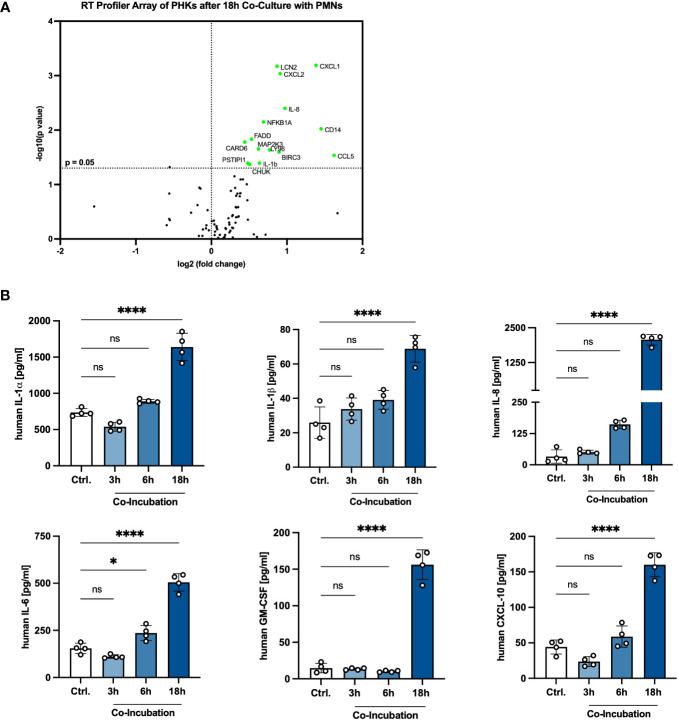
Extended co-incubation activates a proinflammatory state in PHKs. **(A)** Differentiated PHKs were co-incubated with freshly isolated PMNs. After 18h, the expression of 84 different genes was analyzed in PHKs by a RT-Profiler Array (n = 3). Significantly upregulated genes in co-cultured PHKs compared to non-co-cultured PHKs are marked green. **(B)** Secreted factors by PHKs were analyzed after 3h, 6h and 18h of co-incubation with PMNs. Ctrl., non-co-cultured PHKs. Shown is one representative experiment of four independent experiments + SD. Significant differences to the control were analyzed by one-way ANOVA *P < 0.05, **P < 0.01, ***P < 0.001, ****P < 0.0001. One representative experiment of three independent experiments is shown. PHKs, primary human keratinocytes; PMNs, polymorphonuclear neutrophils; RT, real-time. ns, non significant.

Our results show that a crosstalk between PHKs and PMNs boosts the activity of PMNs. Next, we analyzed whether the interaction with PMNs affects, on the other hand, also the PHKs and induces a proinflammatory response in PHKs. For this, we co-cultured PHKs with PMNs for 18h and subsequently analyzed in PHKs the expression of 84 different genes involved in inflammation using an RT-Profiler PCR Array and compared gene expression to 18h non-co-cultured PHKs. Interestingly, we detected significant upregulation of genes associated with immune cell recruitment (CXCL1, CXCL2, IL-8, CCL5), TLR signaling (CD14, LY96), regulation of apoptosis (FADD, CARD6, BIRC3), NFκB signaling (CHUK, NFKB1a, IL-1β) as well as stress response (MAP2K3) in co-cultured PHKs compared to the non-co-cultured PHKs (**Figure 4A**). We further analyzed the induction of a proinflammatory response in PHKs by comparing the secretion of cytokines and chemokines by PHKs co-cultured with PMNs to the non-co-cultured PHKs at different time points using Legendplex analysis. With increasing incubation time, we detected in co-cultured PHKs elevated levels of secreted IL-1α, IL-1β, IL-8, IL-6, GM-CSF, and CXCL-10 compared to the non-co-cultured PHKs (**Figure 4B**). Other analyzed cytokines and chemokines were not significantly induced (**Supplementary Figure 4**). Our results indicate that co-culture with PMNs induce induces a proinflammatory state in PHKs.

### Crosstalk between PHKs and PMNs exacerbates immune responses to *S. aureus* infection

**Figure 5:**
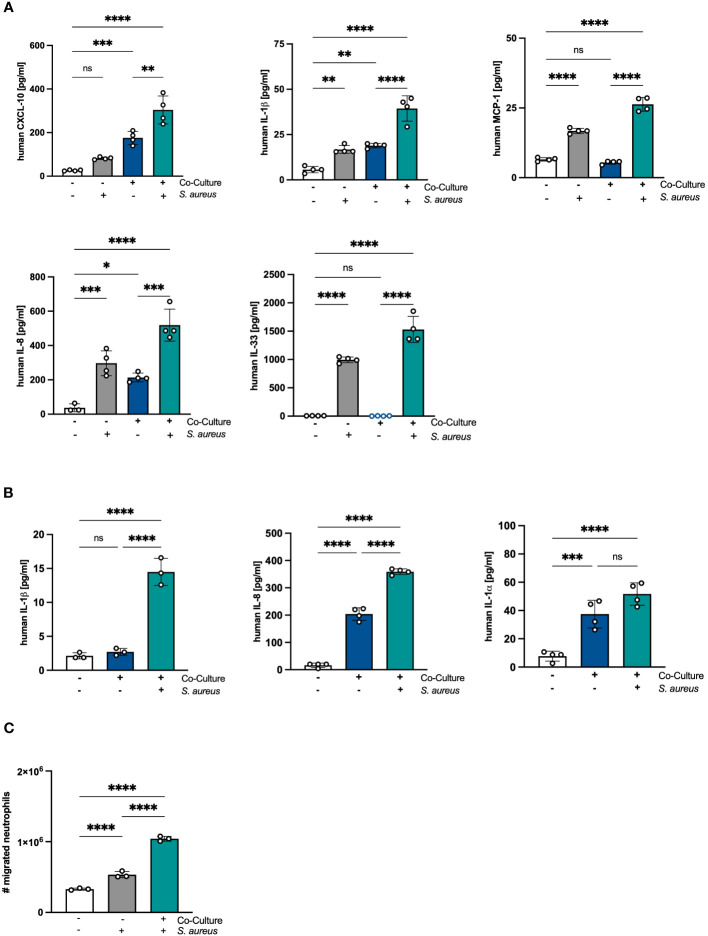
Crosstalk between PHKs and PMNs exacerbates immune responses to *S. aureus* infection. **(A, B)** Differentiated PHKs were co-incubated with freshly isolated PMNs or alone. After 18h, PHKs were infected with *S. aureus* (MOI = 30) for 1.5h or left uninfected. Secreted factors in the PHK well **(A)** and PMN well **(B)** were analyzed by Legendplex analysis. Shown is one representative experiment of four independent experiments + SD. Significant differences between the samples were analyzed by one-way ANOVA *P < 0.05, **P < 0.01, ***P < 0.001, ****P < 0.0001. One representative experiment of three independent experiments is shown. **(C)** Freshly isolated PMNs were seeded into transwell inserts with 3 μm pores and placed above a well containing supernatants of either non-co-cultured or co-cultured PHKs after *S. aureus* infection. After 1h, the number of migrated PMNs were quantified. Shown is one representative experiment of four independent experiments + SD. Significant differences between the samples were analyzed by one-way ANOVA *P < 0.05, **P < 0.01, ***P < 0.001, ****P < 0.0001. One representative experiment of three independent experiments is shown. PHKs = primary human keratinocytes; PMNs, polymorphonuclear neutrophils; MOI, multiplicity of infection; ns, non significant; SD, standard deviation.

Next, we analyzed whether the proinflammatory state in PHKs after co-culture with PMNs under sterile conditions results in an exacerbated response of PHKs after *S. aureus* infection. To explore this, we co-cultured PHKs with PMNs for 18h followed by infection of the PHKs with *S. aureus*. Subsequently, we examined the secretion of proinflammatory cytokines by PHKs using Legendplex analysis. Interestingly, our results revealed that *S. aureus* infection of the PHKs further enhanced the secretion of CXCL10, IL-1β and IL-8 by PHKs in the co-culture. Additionally, *S. aureus* infection induced the secretion of MCP-1, and IL-33 by PHKs, and notably, this induction was significantly higher in co-cultured PHKs compared to non-co-cultured PHKs (**Figure 5A**). The induction of other analyzed cytokine and chemokines were not significantly different between co-cultured and non-co-cultured PHKs (**Supplementary Figure 5A**). Furthermore, *S. aureus* infection of the PHKs in the co-culture significantly enhances the secreted levels of IL-1β, IL-8 and IL-1α in the co-cultured PMNs (**Figure 5B**). Interestingly, the supernatant of co-cultured PHKs and to a lesser extent of non-co-cultured PHKs after *S. aureus* infection significantly enhanced migration of PMNs (**Figure 5C**). These findings demonstrate that the co-culture with PMNs significantly enhances the *S. aureus*-induced inflammatory response in PHKs and PMNs.

### The skin microbiome reduces PMN-mediated inflammation and induces apoptosis in PMNs

**Figure 6:**
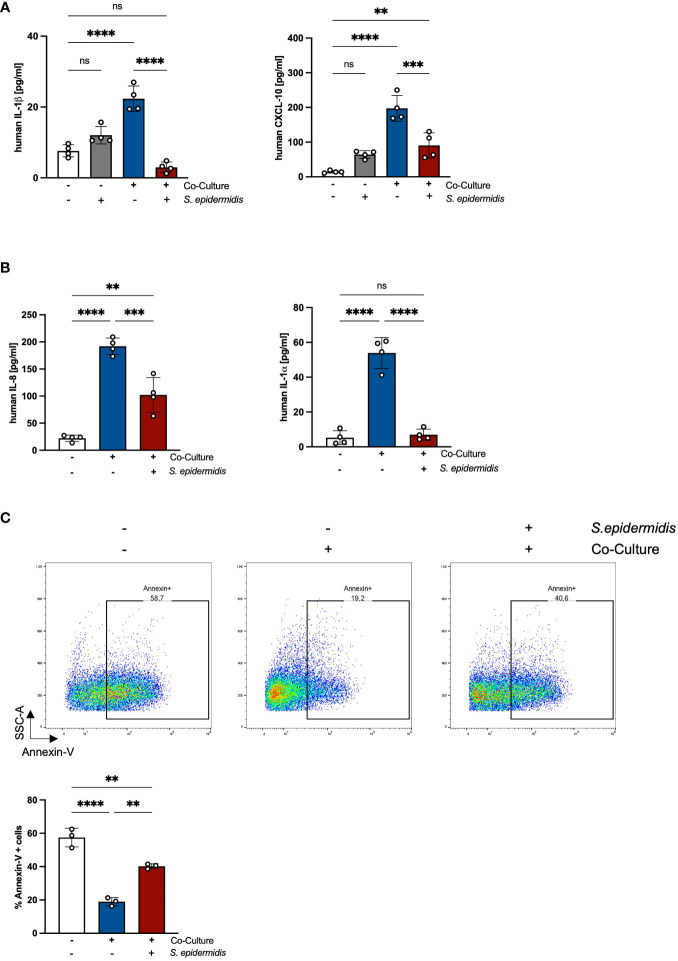
The skin microbiome reduces PMN-mediated inflammation and induces apoptosis in PMNs. **(A, B)** Differentiated PHKs were co-incubated with freshly isolated PMNs or alone. After 18h, PHKs were infected with *S. epidermidis* (MOI = 30) for 1.5h or left uninfected. Secreted factors in the PHK well **(A)** and the PMN well **(B)** were analyzed by Legendplex analysis. Shown is one representative experiment of four independent experiments + SD. Significant differences between the samples were analyzed by one-way ANOVA *P < 0.05, **P < 0.01, ***P < 0.001, ****P < 0.0001. One representative experiment of three independent experiments is shown. **(C)** Freshly isolated PMNs were incubated alone or in co-culture with PHKs. After 18h, PHKs were infected with *S. epidermidis* or left uninfected (MOI = 30) for 1.5h. Apoptosis induction in PMNs was analyzed by Annexin-V staining. Shown is one representative experiment of four independent experiments + SD. Significant differences between the samples were analyzed by one-way ANOVA *P < 0.05, **P < 0.01, ***P < 0.001, ****P < 0.0001. ns, non significant.

Previous results of our group showed that the skin microbiome has a beneficial role in preventing *S. aureus* skin colonization in a non-inflammatory environment (20). Interestingly, the decreased *S. aureus* colonization was accompanied by reduced skin inflammation and PMN recruitment (18). Our results also showed that in an inflammatory environment induced by tape-stripping, skin infiltrating PMNs enhance *S. aureus* skin colonization (18). Based on these results, we hypothesized that the skin microbiome affects PMN-mediated skin inflammation. To test this, we analyzed inflammatory responses in PHKs in the co-culture with PMNs without or upon infection with the skin commensal *S. epidermidis*. Interestingly, we observed a significant decrease in the secreted levels of IL-1β and CXCL-10 by co-cultured PHKs upon *S. epidermidis* infection compared to the cocultured non-infected PHKs (**Figure 6A**). No significant difference between infected and not infected co-cultured PHKs were observed on other analyzed cytokines and chemokines (**Supplementary Figure 6A**). This indicates that the skin microbiome reduces the inflammatory responses in PHKs induced by PMNs. Furthermore, we found that *S. epidermidis* infection of PHKs significantly reduces the secreted levels of IL-8 and IL-1α by PMNs in the co-culture (**Figure 6B**) and significantly enhanced apoptosis induction in PMNs compared to the non-infected and co-cultured PMNs (**Figure 6C**). These results indicate that the skin microbiome reduces PMN-mediated skin inflammation by downregulation of PMN-induced proinflammatory cytokines in PHKs and induction of apoptosis in PMNs.

## Discussion

PMNs are the most abundant type of leucocytes in the human blood and play an important role in the innate immune system (23). Upon inflammation, PMNs are rapidly recruited from the circulation to infection sites where thy provide an effective first-line defense. Several groups have shown that PMNs play a crucial role in clearing *S. aureus* skin infections (15, 16). Here we demonstrate that a crosstalk between skin-infiltrating PMNs and skin-resident PHKs enhances the proinflammatory responses of both cells upon *S. aureus* infection.

PMNs have a relatively short lifespan. They are continually replenished from the bone marrow and released into the bloodstream. In circulation, they actively patrol for infection of tissue and in the absence of an inflammatory stimulus, PMNs undergo spontaneous apoptosis and are cleared by macrophages, maintaining immune balance (10). The lifespan of circulating murine PMNs is estimated to be about 12h (24), however, this can be significantly extended upon inflammation (4). During inflammation, PMNs are rapidly recruited to the affected site. Inflammatory signals promote their survival and activation, enabling them to combat invading pathogens by releasing antimicrobial agents and engaging in phagocytosis (25). However, excessive PMN activation can cause tissue damage, so mechanisms exist to regulate inflammation resolution including the induction of PMN apoptosis and their subsequent clearance by macrophages for immune homeostasis (25).

In their investigation of murine PMN lifespan in various tissues (bone marrow, blood, liver, lung, spleen, intestine, and skin) Ballesteros et al. discovered that the half-life of neutrophils varies depending on the specific tissue they infiltrate into (26). Notably, this study reveals that PMNs quickly adopt a tissue-specific phenotype and transcriptional profile, likely contributing to efficient immune responses against invading pathogens. Interestingly, they showed that the half-life of PMNs was highest in the skin with about 18h.

While several studies have examined the lifespan of PMNs in various tissues of mice, there is limited knowledge regarding the lifespan of PMNs in the human system, especially in the skin. Here, we demonstrate for the first time that the presence of PHKs during co-incubation significantly prolongs the lifespan of PMNs in a human *in vitro* co-culture model. Compared to PMNs cultured alone, co-cultured PMNs exhibit a noticeable delay in apoptosis induction. This extended lifespan is facilitated by the secretion of IL-8, which increases progressively as the incubation time is prolonged. We observed a reversal of this effect when an anti-IL-8 antibody was added to the co-culture. The ability of IL-8 to delay spontaneous apoptosis induction of PMNs is also described by previous studies (27). Interestingly, we did find that depletion of IL-1α, which is also induced in the co-culture, further prevents cell death induction thus indicating that IL-1α is capable of inducing cell death in PMNs. The proapoptotic functions of IL-1α have been described for other cells (28), however, to the best of our knowledge not in PMNs.

Nevertheless, the co-culture with PHKs and IL-8 stimulation did not entirely inhibit PMN apoptosis; instead, it caused a delay in the process, eventually leading to its induction within the co-culture system. Our findings revealed that co-cultured PMNs are activated and released their granules after 18h incubation time. Excessive activation of PMNs can cause tissue damage, so mechanisms exist to regulate inflammation resolution including PMN apoptosis and their subsequent clearance by macrophages for homeostasis (25). Therefore, we hypothesize that the eventual induction of apoptosis in PMNs is required to minimize tissue damage and restore homeostasis.

Interestingly, we found that co-cultured PMNs displayed an elevated activation and responsiveness towards an infectious stimulus in comparison to non-co-cultured or freshly isolated PMNs. This was demonstrated by increased degranulation upon *S. aureus* infection by co-cultured PMNs compared to non-co-cultured PMNs or freshly isolated PMNs. PMNs play a pivotal role in the immune response against *S. aureus* skin infections (16, 29). The reactivity of circulating PMNs towards inflammatory stimuli is intentionally constrained to avoid tissue damage and uphold homeostasis (22). However, this reactivity can be significantly enhanced by the exposure to inflammatory stimuli such as cytokines, chemokines, pathogen- or damage-associated molecular patterns (PAMPs or DAMPs, respectively). The exposure to such stimuli primes the PMNs for enhanced responsiveness towards invading pathogens (22, 30–32). Some studies showed that PMNs primed by proinflammatory cytokines derived from PBMCs exert enhanced killing capacities against *S. aureus* (33, 34). Therefore, we hypothesize that the crosstalk with PHKs contributes to shaping the immune response against *S. aureus* skin infections by priming PMNs for enhanced activation. The co-culture environment seems to provide a beneficial influence on the PMNs, enhancing their ability to combat *S. aureus* skin infections. This finding highlights the significance of cellular interactions and their impact on the immune system’s effectiveness in mounting a robust response against infectious agents.

Moreover, we found that the extended co-incubation not only has a priming effect on PMNs but also induces a proinflammatory state in PHKs. This was characterized by increased secretion of IL-1α, IL-1β, IL-6, IL-8, GM-CSF and CXCL10 by PHKs. CXCL10 and GM-CSF are chemoattractants for other immune cells besides PMNs, such as monocytes, macrophages, T cells, NK cells or dendritic cells (35, 36). This suggests that the extended crosstalk between PHKs and PMNs initiates the recruitment of other immune cells to the skin to facilitate the advance of the immune response. The induction of these proinflammatory mediators is independent on direct cell-to-cell contact but is rather mediated by secreted factors. This finding is supported by previous studies describing the induction of inflammation in PHKs by PMNs (37, 38). For example, Lieu et al. showed that indirect co-culture with PMNs leads to the induction of several pro-inflammatory genes and secretion of IL-8 in HaCaT cells (38). Moreover, Shao et al. showed that exosomes derived from PMNs are able to induce pro-inflammatory gene expression in PHKs (37). Interestingly, although the expression of neutrophilic chemoattractants such as CXCL-1/2 and IL-8 was highly elevated in PHKs after 18h co-culture with PMNs, the supernatant containing these chemoattractants was not able to induce PMN migration. This might be that the concentrations of the respective cytokines were too low for PMN migration. Another reason might be that some factor stops PMN migration as excessive PMN migration and activation can lead to tissue damage.

As PHKs are the main constituents of the epidermis, the outermost layer of our skin, they are constantly exposed to exogenous bacteria such as beneficial skin commensals like *S. epidermidis* or pathogenic bacteria like *S. aureus* and are therefore crucial in the initiation of skin inflammation (2). Here, we found that a previous co-culture with PMNs significantly elevated the level of proinflammatory cytokines and chemokines by PHKs induced by *S. aureus* infection. *S. aureus* is a facultative pathogen which frequently colonizes the skin of atopic dermatitis patients where it actively contributes to inflammation. There is evidence that PMNs are elevated in AD skin and contribute to its pathogenesis (39–41). Furthermore, previous results of our group showed that the PMNs can enhance *S. aureus* skin colonization (18, 19). As a mechanism for this we show that PMNs infiltrating inflamed skin are primed by PHKs for NET formation in response to *S. aureus* infection. The increased presence of PMNs and NETs in the inflamed skin induces oxidative stress in PHKs which results in the secretion of HMGB1, which induces further oxidative stress in PHKs and NET formation in PMNs. Moreover, both NETs and HMGB1 induce the downregulation of epidermal barrier genes in PHKs, thus inducing a skin barrier defect which favors *S. aureus* skin colonization (19). Here, we observed that in addition to promoting skin barrier defects which could enhance *S. aureus* skin colonization, the co-culture with PMNs exacerbates *S. aureus* induced skin inflammation. We believe that the PMNs secrete IL-8 during the co-culture, thereby prolonging their lifespan and becoming activated. The activated PMNs then induce an inflammatory state in PHKs and boost their inflammatory response against *S. aureus*.

The eventual induction of PMN apoptosis in the co-culture might be necessary to not further prolong inflammation. Interestingly, we found that *S. epidermidis* infection of PHKs in the co-culture reduced the proinflammatory responses in PHKs mediated by the co-culture with PMNs. Furthermore, we found that *S. epidermidis* infection of PHKs induced apoptosis in PMNs in the co-culture. Previous work of our group showed that the skin microbiome has a protective role against *S. aureus* skin infections for example by reducing *S. aureus*-mediated skin inflammation and PMN recruitment (18, 20). Our results here further show that the skin commensal bacteria could play a role in preventing PMN-mediated excessive skin inflammation.

In conclusion, here we show for the first time that a crosstalk between PHKs and PMNs in the skin shaped the immune responses against *S. aureus* infections (see graphical abstract). On the one hand, the crosstalk delays the induction of apoptosis in PMNs, prolonging their lifespan and enhancing their activation and responsiveness against *S. aureus*. This effect is achieved through the release of IL-8 rather than direct cell-to-cell contact. However, it is important to note that prolonged incubation with PMNs also lead to inflammatory responses in keratinocytes. This inflammatory response is further exacerbated in the presence of *S. aureus*. Interestingly, the skin commensal *S. epidermidis* reduces the PMN-mediated skin inflammation in PHKs and induces apoptosis in activated PMNs which indicates a beneficial role of the skin microbiome in preventing excessive inflammation.

## Data availability statement

The original contributions presented in the study are included in the article/**Supplementary Material**. Further inquiries can be directed to the corresponding author.

## Ethics statement

The studies involving humans were approved by University of Tübingen Ethics committee. The studies were conducted in accordance with the local legislation and institutional requirements. The participants provided their written informed consent to participate in this study.

## Author contributions

BS: Conceptualization, Funding acquisition, Project administration, Supervision, Writing – original draft. JF: Data curation, Formal analysis, Investigation, Methodology, Writing – original draft.
