# Supplementary material for: Crosstalk between keratinocytes and neutrophils shapes skin immunity against S. aureus infection
Source: Front Immunol. 2024 Feb 16;15:1275153. doi: 10.3389/fimmu.2024.1275153 (PMC10911042; doi:10.3389/fimmu.2024.1275153)
Supplement: Supplementary file 1 [file DataSheet_1.pdf]

Supplementary Material

Supplementary Figure 1

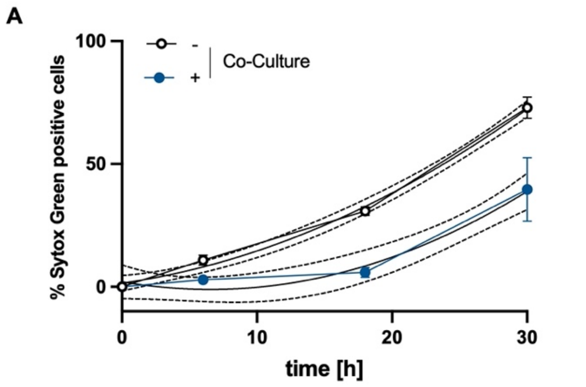

Sytox Green positive cells	Time [h] Interpolated		Delay [h]
	No Co-Culture	Co-Culture	
50%	23.8 h	33.1 h	9.3 h

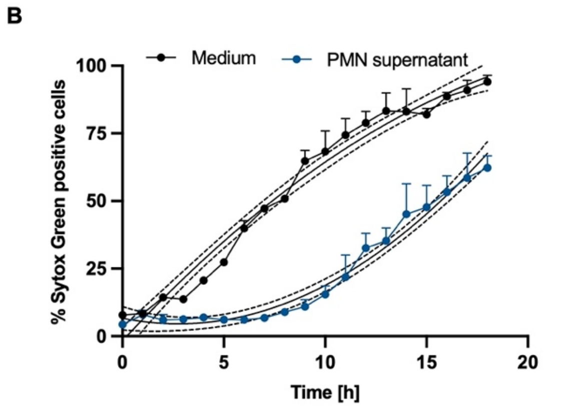

Sytox Green positive cells	Time [h] Interpolated		Delay [h]
	Medium	PMN Supernatant	
50%	7,39 h	15,7 h	8.31 h

Supplementary Figure 1: Calculation of the delay of cell death induction using interpolation A&B: Calculation of cell death delay between co-cultured and non-co-cultured PMNs (**A**) and PMNs incubated in medium or PMN supernatant (**B**) using interpolation. Shown are the mean of four different experiments + SEM. PMNs = polymorphonuclear neutrophil; SEM = standard error of the mean

Supplementary Figure 2

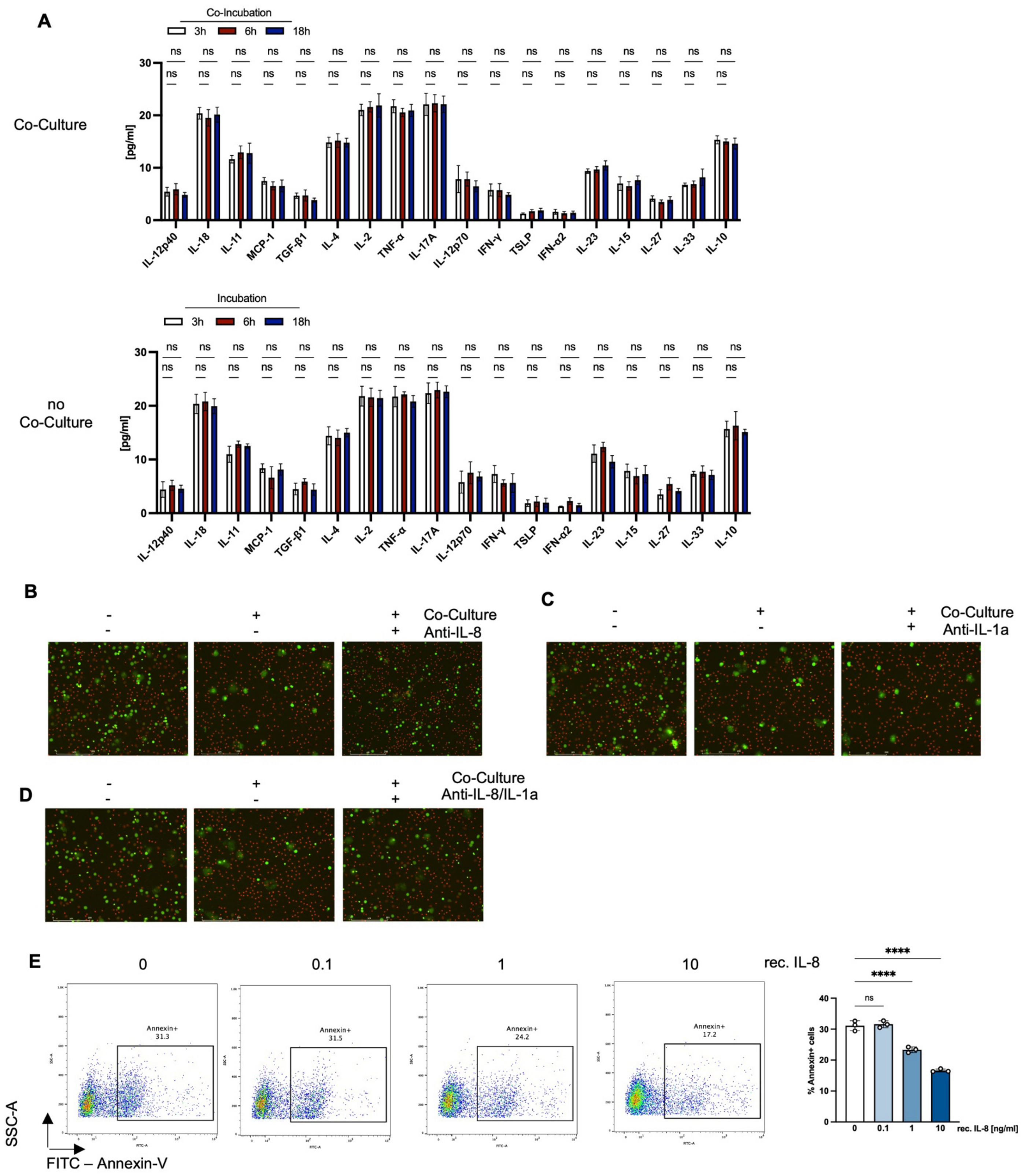

Supplementary Figure 2: Legendplex analysis, viability analysis of PMNs after IL-8 and IL-1α blockade. A: Non-significantly induced cytokines and chemokines in PMNs co-cultured with PHKs or alone for different time points. Non-co-cultured PMNs served as control. Shown is one representative experiment of four different experiments + SD Significant differences to the control were analyzed by two-way ANOVA *P < 0.05, **P < 0.01, ***P < 0.001, ****P < 0.0001. **B-D:** PMNs were co-cultured with PHKs for 18h in the presence or absence of anti-IL-8 (**B**), anti-IL-1a (**C**), or both (**D**). After 18h, cell viability was analyzed by SYTOX Green staining. Representative pictures are shown. **E:** freshly isolated PMNs were stimulated with different concentrations of recombinant human IL-8. After 18h, apoptosis induction was analyzed by Annexin-V staining and flow cytometry. Shown is one representative experiment of three independent experiments + SD. Percentage of apoptotic cells was compared to unstimulated PMNs by one-way ANOVA *P < 0.05, **P < 0.01, ***P < 0.001, ****P < 0.0001. PMN = polymorphonuclear neutrophils; PHK = primary human keratinocyte; SD = standard deviation

Supplementary Figure 3

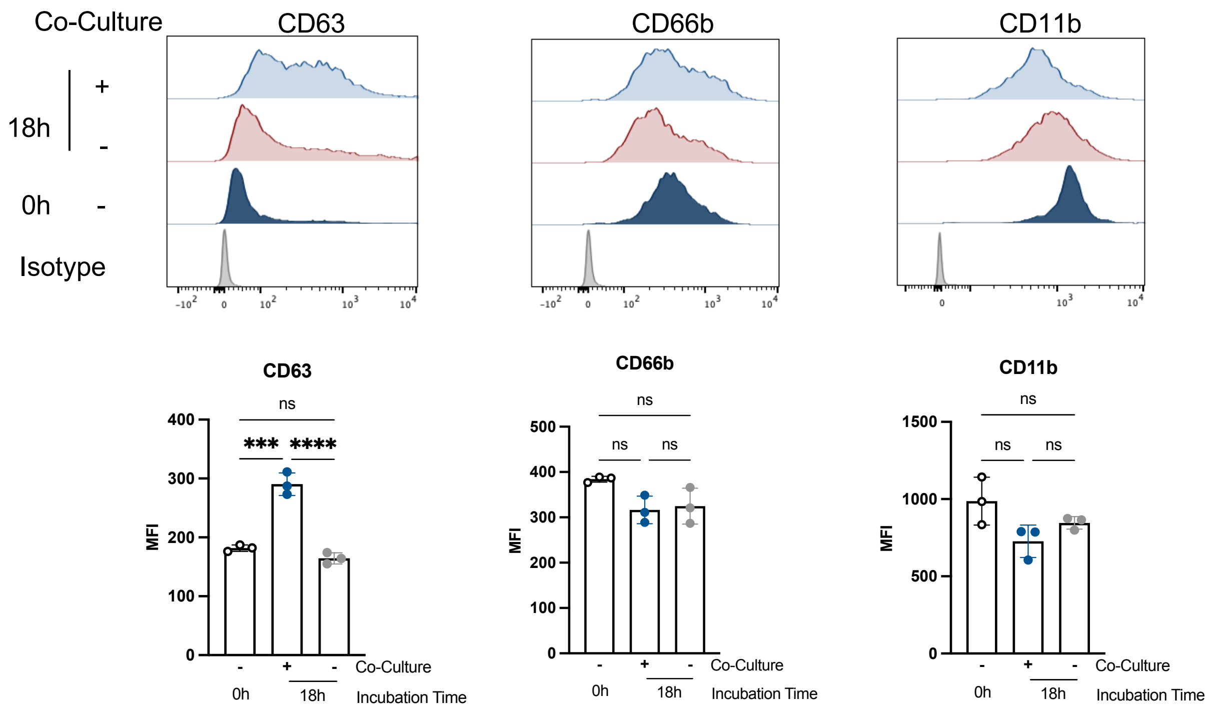

Supplementary Figure 3: Surface expression of activation markers on co-cultured and non-co-cultured PMNs after 18h. Histograms and quantification of surface expression of CD63, CD66b, CD11b on freshly isolated, 18h co-cultured and non-co-cultured PMNs. Shown is one representative experiment of three independent experiments + SD. Significant differences between the samples were analyzed by one-way ANOVA $*P < 0.05$, $**P < 0.01$, $***P < 0.001$, $****P < 0.0001$. PMNs = polymorphonuclear neutrophils; SD = standard deviation

Supplementary Figure 4

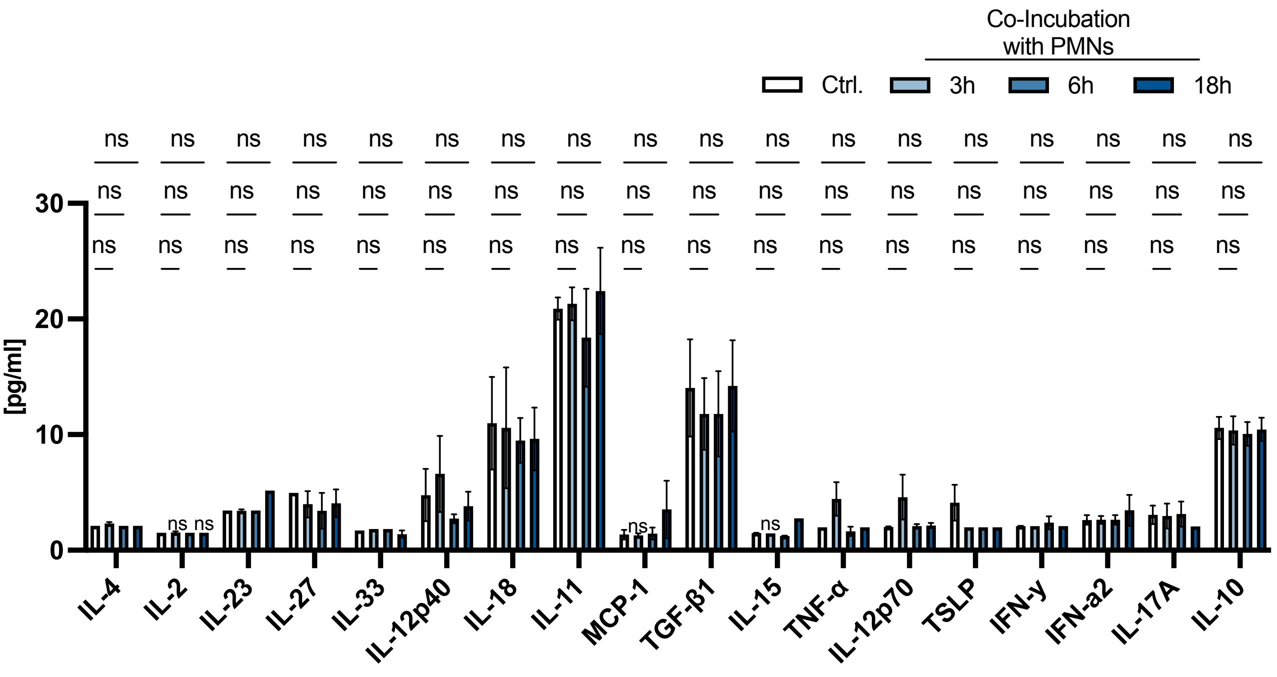

Supplementary Figure 4: Non-significantly induced cytokines in PHKs co-incubated with PMNs. To see, if proinflammatory responses upon *S. aureus* infection are affected by the co-culture, differentiated PHKs were co-incubated with PMNs for 3h, 6h and 18h, Secreted cytokines were analyzed by Legendplex. Uninfected PHKs were used as control. Shown is one representative experiment of four independent experiments + SD. Significant differences to the control were analyzed by one-way ANOVAs. *P < 0.05, **P < 0.01, ***P < 0.001, ****P < 0.0001. PHKs = primary human keratinocytes; PMNs = polymorphonuclear neutrophil; *S. = staphylococcus aureus;* SD = standard deviation

Supplementary Figure 5

A

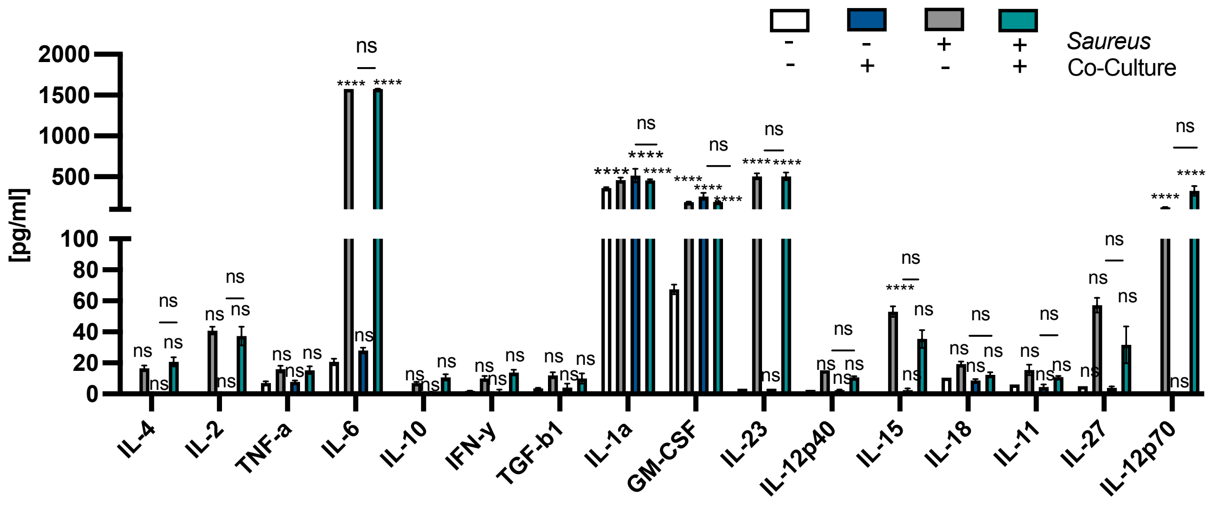

B

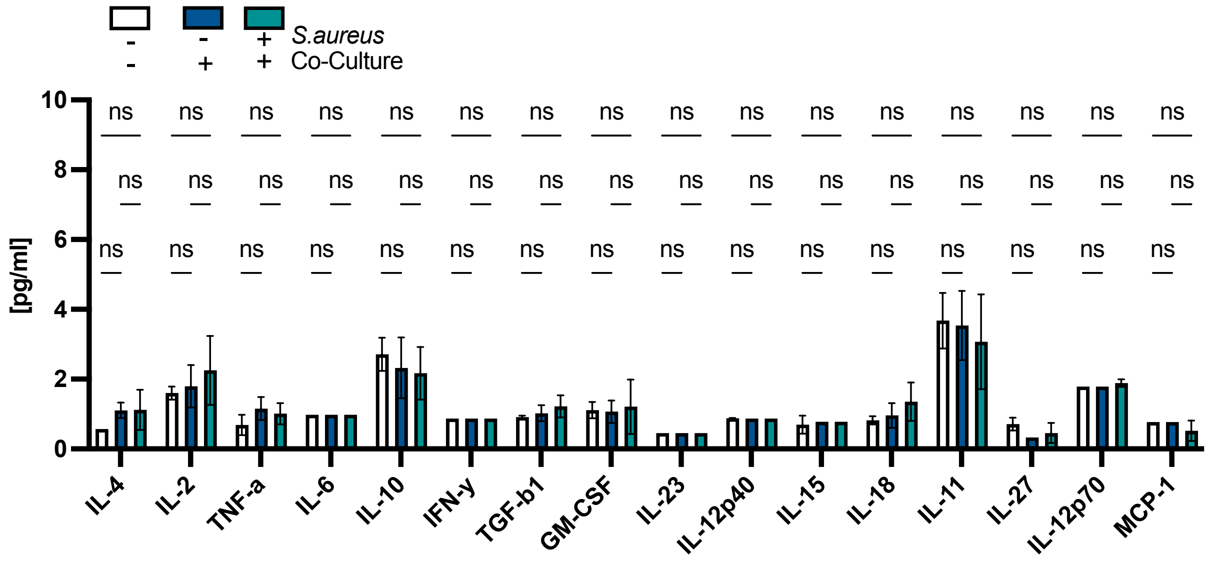

Supplementary Figure 5: Non-significantly induced cytokines in PHKs and PMNs after *S. aureus* infection in the co-culture. **A:** To see, if proinflammatory responses upon *S. aureus* infection are affected by the co-culture, differentiated PHKs were co-incubated with PMNs for 18h and then infected with *S. aureus* or left uninfected for 1.5h. Secreted cytokines were analyzed by Legendplex. Uninfected PHKs were used as control. Shown is one representative experiment of four independent experiments + SD. Significant differences to the control were analyzed by two-way ANOVA *P < 0.05, **P < 0.01, ***P < 0.001, ****P < 0.0001. **B:** PMNs were either co-cultured with PHKs or alone for 18h and subsequently, PHKs were infected with *S. aureus* (MOI = 30). Secreted cytokines were analyzed by Legendplex analysis. Shown is one representative experiment of four independent experiments + SD. Significant differences between the samples were analyzed by two-way ANOVA *P < 0.05, **P < 0.01, ***P < 0.001, ****P < 0.0001. PHKs = primary human keratinocytes; PMNs = polymorphonuclear neutrophils; *S.* = *Staphylococcus;* SD = standard deviation

Supplementary Figure 6

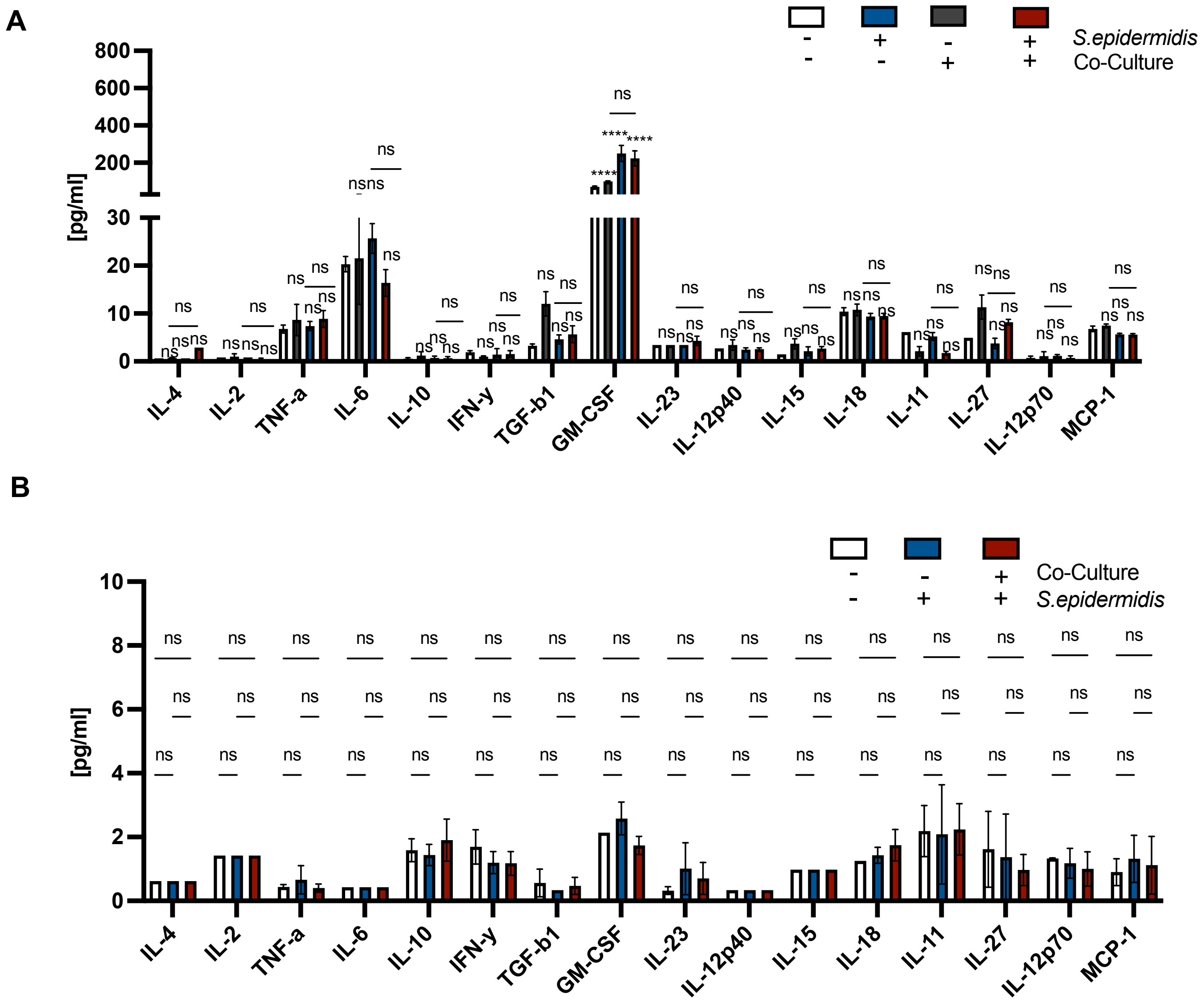

Supplementary Figure 6: Non-significantly induced cytokines in PHKs and PMNs after *S. epidermidis* infection in the co-culture. A: To see, if proinflammatory responses upon *S. epidermidis* infection are affected by the co-culture, differentiated PHKs were co-incubated with PMNs for 18h and then infected with *S. epidermidis* (MOI = 30) or left uninfected for 1.5h. Secreted cytokines were analyzed by Legendplex. Uninfected PHKs were used as control. Shown is one representative experiment of four independent experiments + SD. Significant differences between the samples were analyzed by two-way ANOVA *P < 0.05, **P < 0.01, ***P < 0.001, ****P < 0.0001. **B:** PMNs were either co-cultured with PHKs or alone for 18h and subsequently, PHKs were infected with *S. epidermidis* (MOI = 30). Secreted cytokines were analyzed by Legendplex analysis. Shown is one representative experiment of four independent experiments + SD. Significant differences between the samples were analyzed by two-way ANOVA *P < 0.05, **P < 0.01, ***P < 0.001, ****P < 0.0001. PHKs = primary human keratinocytes; PMNs = polymorphonuclear neutrophils; *S.* = *Staphylococcus;* MOI = multiplicity of infection; SD = standard deviation
